# Motor performance and back pain in children and adolescents: a systematic review and meta-analysis protocol

**DOI:** 10.1186/s13643-020-01468-6

**Published:** 2020-09-14

**Authors:** Matias Noll, Niels Wedderkopp, Carolina Rodrigues Mendonça, Per Kjaer

**Affiliations:** 1 Instituto Federal Goiano, Ceres, Brazil; 2 Postgraduate Program in Health Sciences, Faculty of Medicine, Federal University of Goiás, Goiânia, Brazil; 3 Department of Sports Science and Clinical Biomechanics, University of Southern Denmark, Campusvej 55, Odense, Denmark; 4 Sports Medicine Clinic, Orthopedic Department, Hospital of Lillebaelt, Odense, Denmark; 5 Health Sciences Research Centre, UCL University College, Odense, Denmark

**Keywords:** Back pain, Pain, Teenagers, Psychomotor performance, Protocol, Systematic review

## Abstract

**Background:**

The relationship between motor performance and back pain in children and adolescents remains unclear. This article describes the protocol for a systematic review to summarize the knowledge about the association between motor performance, such as agility, flexibility, balance, strength, muscle endurance, and cardiorespiratory fitness, and back pain. Thus, our aim is to identify the influence of motor performance on back pain among children and adolescents.

**Methods:**

Two independent researchers will search MEDLINE, Scopus, Embase, SPORTDiscus, and CINAHL databases, with no period or language restrictions. We will include cross-sectional, cohort, case-control, and controlled clinical trial studies based on the following criteria: (a) participants from 6 to 19 years of age, (b) assessment of motor performance, (c) assessment of back pain, and (d) report measures of associations between motor performance and back pain. Study quality and risk of bias will be assessed using an adapted version of the Downs and Black instrument. Grading of Recommendations, Assessment, Development, and Evaluations will be used to assess the strength of the body of evidence. Meta-analyses of association measures will be performed for each type of motor performance, separately for different study types. The results will be reported using forest to show the pooled effect of findings and funnel plots to assess precision of the data. If studies are not homogeneous, results from the meta-analyses will not be reported. Associations will then be synthesized descriptively using a pragmatic approach.

**Discussion:**

This systematic review will provide critical insights into the association between motor performance and back pain among children and adolescents; this information may help support clinical practice guidelines as well as public health programs.

**Ethics and dissemination:**

Protocol was written according to the Preferred Reporting Items for Systematic reviews and Meta-Analyses (PRISMA).

**Systematic review registration:**

PROSPERO CRD42020178496

## Background

Back pain (BP), defined as “pain within the cervical, thoracic and/or lumbar areas” [1–3], is a significant public health problem [1–7]. Recent studies [8–10] suggest that the prevalence of BP, including acute, chronic, specific, and non-specific conditions, has been increasing among adolescents; it can cause a range of functional limitations that may persist into adulthood [11–13]. Therefore, the causes of BP among adolescents should be investigated [14]. Studies have shown that income level [9], sex [15], psychological and social factors [16], sedentary behavior [17], sleep problems [18], and cardiovascular risks [19] may be associated with BP among children and adolescents. Several studies have focused on the relationship between physical activity [20] or sports variables (e.g., the type of sport and frequency and intensity of exercise) [21–25] and BP.

The relationship between motor performance and BP remains unclear. Motor performance components are essential to psychological and social development in children and adolescents [26] and have been recognized as an important determinant of current and future health status [27, 28]. Recent reviews [1, 2, 29] evaluating potential risk factors for BP pain in children and young adults reported that there were few studies on components of motor performance, including agility, strength in the lower extremity, hand grip strength, and cardiorespiratory fitness, and that these relationships are unclear. Motor performance is an important health parameter that is positively associated with physical activity [30]; however, it may also be related to injuries [31].

Pain during adolescence is an important predictor of pain in adulthood [32]. Therefore, an adequate assessment in children and adolescents is fundamental to help researchers and health professionals better understand BP and associated risk factors to improve health promotion programs and interventions. To the best of our knowledge, there is a lack of understanding about the relation between the levels of motor performance, such as agility, flexibility, balance, strength, muscle endurance, and cardiorespiratory fitness and BP, as well as the particularities for sex and age groups. Thus, the present systematic review will focus on the following research question: “How could different levels of motor performance influence BP in young people?”

Our study aims to identify the influence of motor performance on BP in children and adolescents and to investigate the differences between sexes and age groups. Our main hypothesis is that higher levels of motor performance are related to reduced BP. Understanding these factors may enable health professionals to perform more targeted and effective health promotion interventions.

## Methods

### Protocol and registration

This systematic review will be conducted according to the Preferred Reporting Items for Systematic Reviews and Meta-analyses (PRISMA) guidelines [33] for the identification, screening, eligibility, and inclusion (Additional file 1). Our review follows the Population, Exposure, Comparator, and Outcome (PECO) structure, which is explained in the recommended notification items for systematic reviews [34]. Accordingly, “P” represents children and adolescents, “E” represents “motor performance,” “C” represents “sex and age group,” and “O” represents “BP variables.”

The current protocol is registered in the International Prospective Register of Systematic Reviews (PROSPERO) [35] (Registration number CRD42020178496). Any amendments made to this protocol when conducting the study will be reported in PROSPERO and in the final manuscript.

### Search strategy and eligibility criteria

In fall 2020, one researcher (MN) will search the MEDLINE, Scopus, Embase, SPORTDiscus, and CINAHL databases, with no period and language restriction. The detailed search strategy is presented in Table 1. Disagreement with respect to the searches will be discussed and resolved. In addition, search strategy will be supplemented by (a) citation tracking in the reference list of the included studies and relevant systematic reviews and (b) via Google Scholar searches.

**Table 1:** Search strategy

1 = “back pain” OR backache OR backaches OR “back ache” OR “back aches” OR "spinal pain" OR “spine pain” OR “neck pain” OR “neck ache” OR “neck aches” OR “cervical pain” OR lumbago OR "lumbar pain" 2 = “Motor performance” OR “Motor performances” OR “fitness performance” OR “fitness performances” OR “fitness assessment” OR “movement assessment” OR “motor assessment” OR “psychomotor performance” OR “psychomotor performances” OR “motor fitness” OR Strength OR “abdominal muscular endurance” OR Flexibility OR Balance OR Agility OR Jump OR “cardiorespiratory fitness” OR “cardiorespiratory performance” OR “aerobic endurance” OR “anaerobic endurance” OR “aerobic fitness” OR “anaerobic fitness” 3 = child OR children OR adolescent OR adolescents OR minors OR youth OR youths OR juvenile OR teen OR teens OR teenager OR teenagers OR childhood OR adolescence OR schoolchild OR “school child” OR schoolchildren OR “school children” OR young OR youngs 4 = 1 AND 2 AND 3	

We will include cross-sectional, cohort, case-control, and controlled clinical trial (data from control groups) studies. The inclusion criteria will be (a) participants aged 6 to 19, (b) assessment of motor performance, (c) assessment of BP, and (d) report measures of associations between motor performance and BP, or data that makes it possible to estimate associations.

“Motor performance” will be defined as “an observable action or behavior that can be measured (rated) by an observer” [36] which include the evaluation of agility, flexibility, balance, strength, muscle endurance, and cardiorespiratory fitness. BP is defined as “pain within the cervical, thoracic and/or lumbar areas including acute, chronic, specific, and non-specific conditions” [1–3].

The exclusion criteria will be as follows: (a) studies with incomplete data or review articles, opinion articles, and case reports; (b) non-quantitative measures in the assessment of motor performance; (c) specific populations that included athletes, hospitalized adolescents, diseased people, disabled people, amputees, or pregnant and lactating women; (d) mixing of athletes with non-athletes, unless the data were reported separately or could be calculated from the data provided; and (e) mixing of children and adolescents with other group ages, unless the children’s and adolescents’ data were reported separately or could be calculated from the data provided.

### Training of the reviewers

The authors who will participate in eligibility assessments are being trained regarding the study inclusion/exclusion criteria and will complete practice eligibility assessments on 50 test abstracts before starting to code articles. The authors also will train in performing risk of bias instruments on five not-included articles, as well as standardized analyses using Mendeley and Rayyan software.

### Review process

**Fig. 1:**
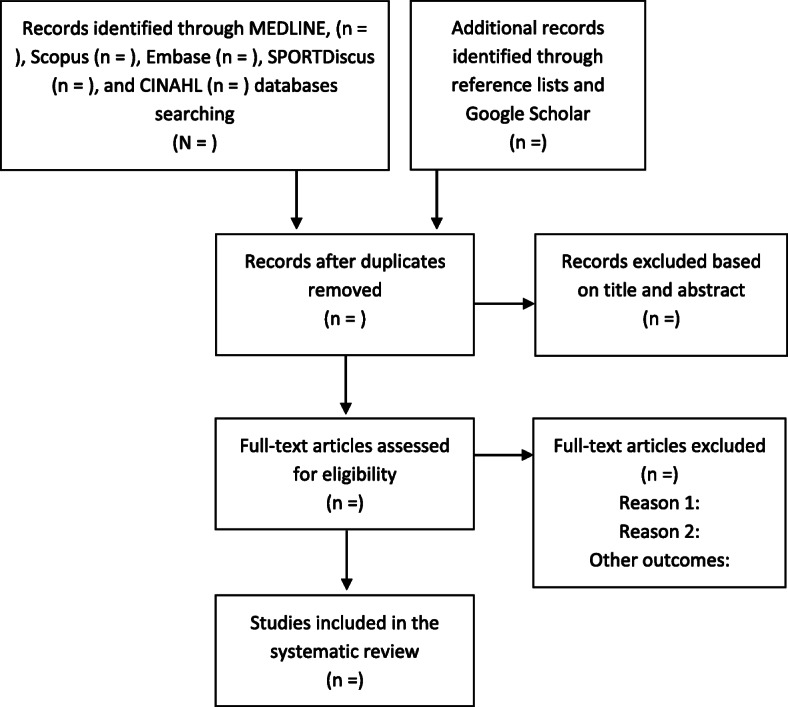
Flow diagram of the selection criteria for the study

After executing the search strategy, articles will be collated, and duplicates will be removed using the Mendeley Software. Then, two reviewers (MN and CRM) will independently screen the titles and abstracts of all articles identified in the literature search for inclusion. Disagreement regarding inclusion will be discussed and resolved by a third reviewer (PK or NW). The screening process will be performed for both reviewers using Rayyan Software [37]. Inter-rater reliability for individual component ratings will be determined by calculating the percentage of agreement and Cohen’s Kappa coefficient [38]. The remaining articles will be read in full and evaluated to determine their eligibility based on the inclusion and exclusion criteria. Finally, the eligible articles will be included in the systematic review. In addition, the reference lists of included articles will be searched to identify additional studies missed by database searches. The flow chart for this systematic review is shown in Fig. 1.

### Data extraction and quality assessment

From the selected articles, the following data will be extracted: authors, publication year, ethical approval, conflict of interest, sample size, sex, age, location/country, study design, main and secondary outcomes, instruments for measurement, major findings, and association values. Regarding BP, we will record the pain definition, time since onset, prevalence, frequency, intensity, and site(s). These characteristics of the articles included in the systematic review will be presented in Additional file 2 and Tables 2 and 3. If during the process, needs to include more information arise, more columns will be included in these Tables.

**Table 2:** Summary of the articles included in the systematic review

Characteristics	Number of studies (%)
Publication year	
Prior to 1990	
1991–2000	
2001–2010	
2011–2020	
Study design	
Cross-sectional	
Longitudinal	
Intervention	
Region/country	
Region A	
Region B	
Region C	
Region D	
Region E	
Region F	
Sex	
Male sex only	
Female sex only	
Mixed	
Sample size	
< 100	
100–1000	
> 1000	
Motor performance variables	
Outcome A	
Outcome B	
Outcome C	
Outcome D	
Outcome E

**Table 3:** Quantitative characteristics of the articles included in the systematic review and results of the association

Author/year	Design/country	Subjects, *N* (% male)	Age, (years/range)	Motor performance test	Back pain definition	Region pain/period	Pain prevalence	Main result (motor performance × pain)
.	.	.	.	.	.	.	.	.

Risk of bias will be assessed using the 27-item Downs and Black instrument [39]. For observational studies, not all items on the Downs and Black checklist are generally applicable; therefore, a subset of 19 questions (questions 1–3, 5–7, 9–12, 15–18, 20, 21, 25–27) will be used. A summary quality score (0–19 points) for each manuscript will be calculated by expressing the number of compliant items as a percentage of the total. Score above 70% will be used to define low risk of bias [39].

The Grading of Recommendations, Assessment, Development, and Evaluations (GRADE) evidence system will be used to assess the quality of evidence [40]. For each study, quality of evidence is ultimately assigned one of four grades: high quality, moderate quality, low quality, or very low quality [41] (Additional file 2).

We will also report whether the authors of the included studies stated potential conflicts of interest and gave information on ethical approval [42]. Data will be extracted and assessed by two independent reviewers (MN and CRM), and disagreements will be resolved by a third reviewer (PK or NW). When relevant data are not available in the manuscript, a researcher (MN) will contact the authors directly to fill these gaps.

Our systematic review is focused on the analysis of published articles (secondary data), and it does not require ethics approval. After the conclusion, the authors will submit the systematic review for publication in a peer-reviewed journal.

### Evidence synthesis and statistical analyses

We will present an overview of the available studies, describe the prevalence of the various types of BP, and report motor performance indicators in participants with and without BP. Absolute differences in motor performance and appropriate measures of association (relative risks, odds ratios, or diagnostic measures) will be compared.

Regarding these data, we plan to perform a meta-analysis if there are sufficient studies with low risk of bias [39, 43]. Firstly, we will analyze the mean difference including 95% confidence intervals in motor performance levels among children and adolescents with BP, considering the differences between girls and boys, and age groups, by either adjusting for or stratifying by sex and age groups. This will be reported separately for cross-sectional and longitudinal studies. Secondly, we will pool findings into random-effects meta-analysis and report these results using forest to show the pooled effect of findings and funnel plots to assess precision of the data [43]. Thirdly, the percentage of variability attributable to heterogeneity will be estimated using the *I*^2^ test. *I*^2^ values of < 40, 40–60, 60–90, and > 90% correspond, respectively, to “not important,” “moderate,” “substantial,” and “considerable” levels of heterogeneity as described by the Cochrane collaboration [43]. Meta-analysis will be performed in the STATA 16.0 Software (Stata Corporation, College Station, TX, EUA) at 95% confidence interval. Publication bias will be assessed by visual inspection of a funnel chart. When interpreting the pooled estimates from each meta-analysis, the GRADE evaluation of each included study will be used to rate the overall quality of evidences.

We will present one meta-analysis for each motor performance test (agility, flexibility, balance, strength, muscle endurance, and cardiorespiratory fitness) as well as separately for cross-sectional and longitudinal studies. If meta-analyses are not appropriate, data will be synthesized descriptively using the forest plots and a pragmatic descriptive approach.

## Discussion

To the best of our knowledge, this will be the first systematic review that aims to summarize studies on the relationship between motor performance and BP among children and adolescents; it will determine if there are any associations and, if so, how strong they are. In this study, the results from the cross-sectional studies can only provide information about association and not causality, whereas longitudinal studies can indicate the direction of the influences.

It is crucial to identify how motor performance and BP are associated in this age group, because BP and its harmful impacts increase during transition from childhood to adolescence. First, it is crucial to understand the risk factors and detect groups of frail children, who are particularly susceptible for developing BP [3] to prevent negative consequences. Second, because motor performance is trained in physical education classes, our result may provide valuable quantitative information to these educational and health professionals, which in turn could increase the quality of their physical education classes, avoiding unwanted BP.

Some limitations may be expected. First, various measurements and tools might lead to varying levels of motor performance outcomes. Second, the lack of clarity in the definition of BP might complicate the comparisons. Third, if no study meets our eligibility criteria, the review will be reported as an “empty review.” However, even an “empty review” is relevant and might help stimulate appropriate future investigations [44].

Strengths of this study include assessment of a wide motor performance scope, absence of language and year restrictions, evaluation of quality and risk of bias analysis, and reporting of conflict of interest and ethical approval, all of which are often omitted from reviews. This systematic review will provide critical insights into the associations between motor performance and BP among children and adolescents that may be important for supporting the development of clinical practice guidelines and public health programs.

## Supplementary information

**Additional file 1:.** PRISMA-P (Preferred Reporting Items for Systematic Review and Meta-Analysis Protocols) 2015 checklist. Recommended items to address in a systematic review protocol.

**Additional file 2:.** Methodological quality assessment and strength of evidence.

## Data Availability

Not applicable.

## Abbreviations

GRADE: Grading of Recommendations, Assessment, Development, and Evaluations
PRISMA: Preferred Reporting Items for Systematic Reviews and Meta-analyses
PROSPERO: International Prospective Register of Systematic Reviews
PECO: Population, Exposure, Comparator, and Outcome
