# Supplementary material for: Motor performance and back pain in children and adolescents: a systematic review and meta-analysis protocol
Source: Syst Rev. 2020 Sep 14;9:212. doi: 10.1186/s13643-020-01468-6 (PMC7491087; doi:10.1186/s13643-020-01468-6)
Supplement: Supplementary file 2 — Additional file 2:. Methodological quality assessment and strength of evidence. [file 13643_2020_1468_MOESM2_ESM.docx]

**Additional file 2.** Methodological quality assessment and strength of evidence.

| **Study (year)** | **Conflict**  **of interest** | **Ethical approval** |  | **Downs and Black checklist** | | | | | | | | | | | | | | | | | | | |  | **GRADE** |
| --- | --- | --- | --- | --- | --- | --- | --- | --- | --- | --- | --- | --- | --- | --- | --- | --- | --- | --- | --- | --- | --- | --- | --- | --- | --- |
|  |  |  |  | A | B | C | D | E | F | G | H | I | J | L | K | M | N | O | P | Q | R | S | Score |  |  |
| Study A (year) | Yes | Yes |  | 1 | 0 | 0 | 1 | 1 | 1 | 1 | 1 | 1 | 1 | 1 | 1 | 1 | 1 | 1 | 0 | 0 | 1 | 1 | 79% |  | 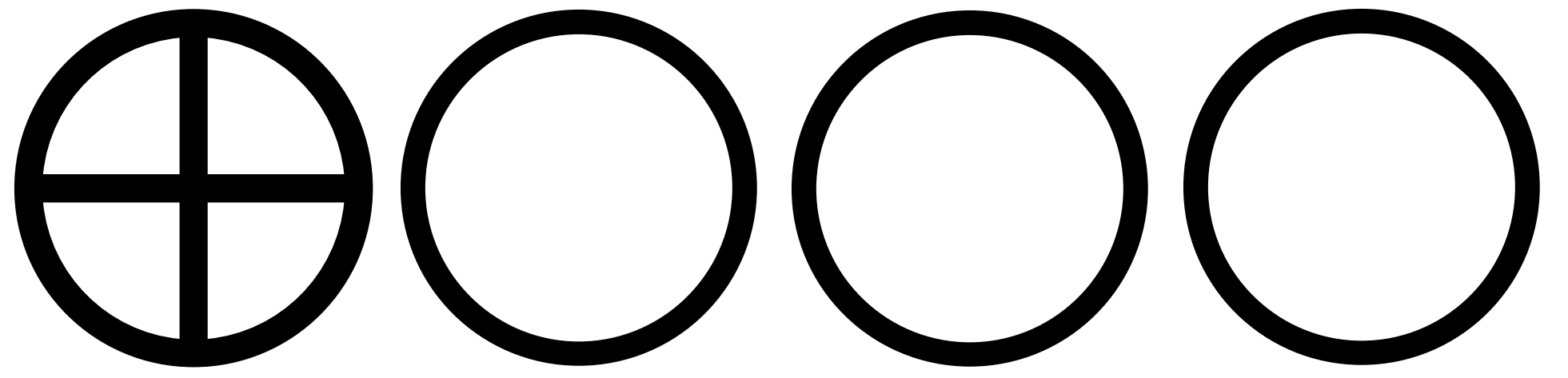 |
| Study B (year) |  |  |  |  |  |  |  |  |  |  |  |  |  |  |  |  |  |  |  |  |  |  |  |  | 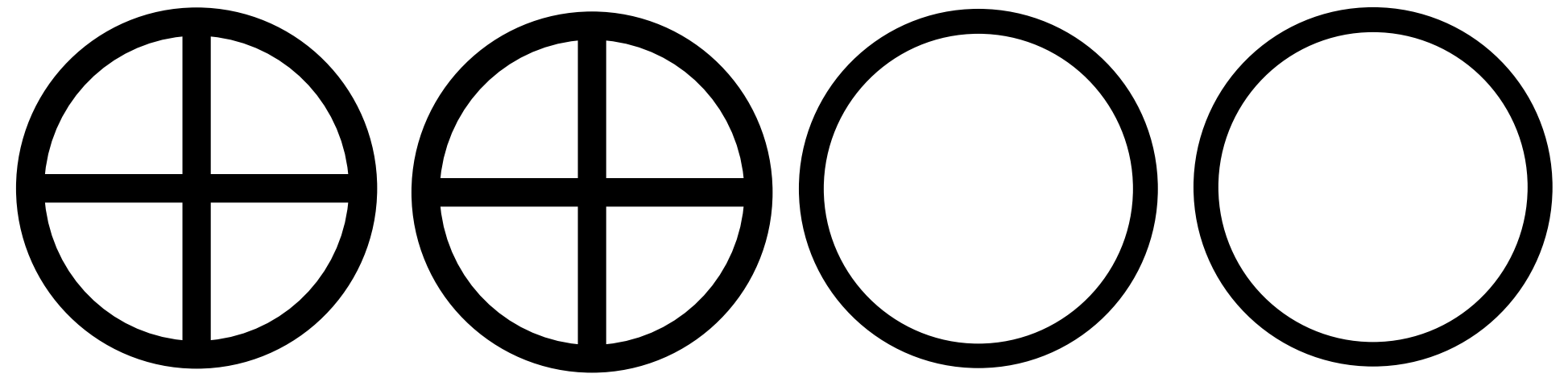 |
| Study C (year) |  |  |  |  |  |  |  |  |  |  |  |  |  |  |  |  |  |  |  |  |  |  |  |  | 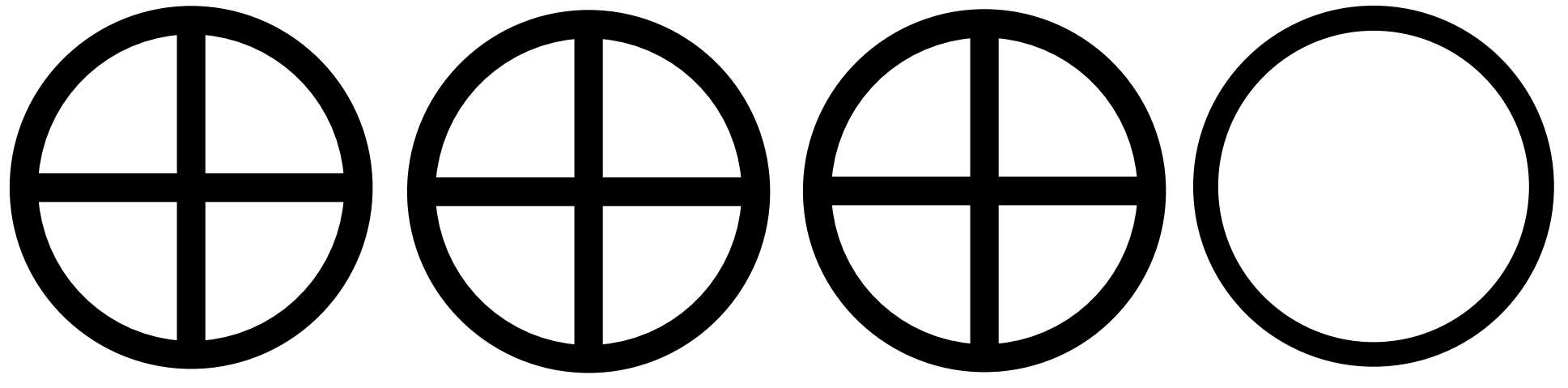 |
| Study D (year) |  |  |  |  |  |  |  |  |  |  |  |  |  |  |  |  |  |  |  |  |  |  |  |  | 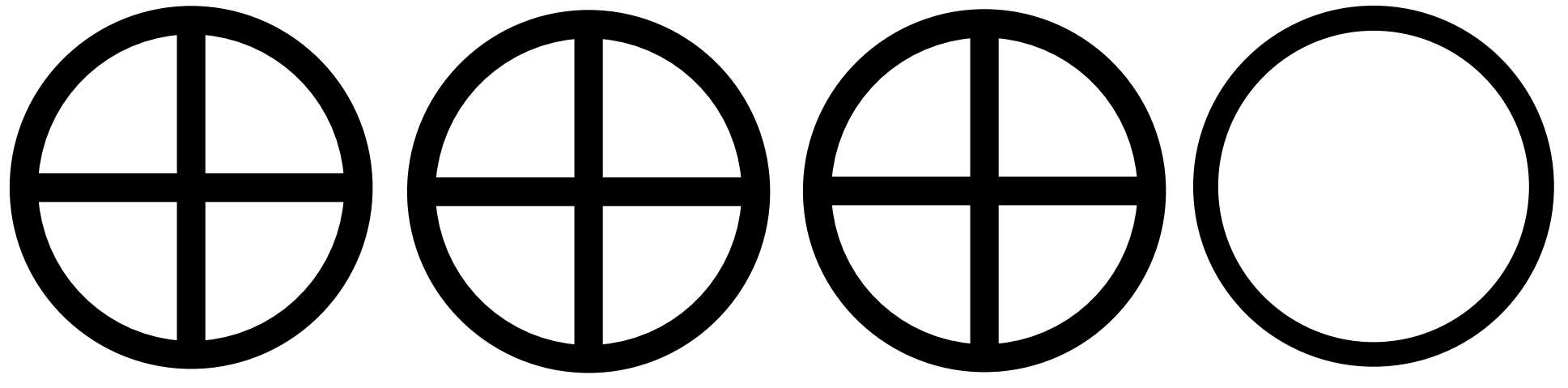 |

Downs and Black checklist: A, objective clearly stated; B), main outcomes clearly described; C) patient characteristics clearly defined; D), distribution of principal confounders clearly described; E) main findings clearly defined; F) random variability in estimates provided; G) lost to follow-up described; H) probability values reported; I) sample target representative of population; J) sample recruitment representative of population; K) blind those measuring the main outcomes; L) study based on “data dredging,” if applied; M) analyses adjust for different lengths of follow-up; N) statistical tests used appropriately; O) primary outcomes valid/reliable; P) sample recruited from the same population; Q) adequate adjustment for confounding; R) losses of sample to follow-up taken into account; and S) sufficient power to detect a clinically important effect.

GRADE: Grading of Recommendations, Assessment, Development and Evaluations. One filled circle: very low quality; two filled circles: low quality; three filled circles: moderate quality; four filled circles: high quality.

* Not mentioned.
